# The Comparative Abilities of a Small Laccase and a Dye-Decoloring Peroxidase From the Same Bacterium to Transform Natural and Technical Lignins

**DOI:** 10.3389/fmicb.2021.723524

**Published:** 2021-10-18

**Authors:** Thu V. Vuong, Rahul Singh, Lindsay D. Eltis, Emma R. Master

**Affiliations:** ^1^Department of Chemical Engineering and Applied Chemistry, University of Toronto, Toronto, ON, Canada; ^2^Department of Microbiology and Immunology, BioProducts Institute, The University of British Columbia, Vancouver, BC, Canada; ^3^Genome British Columbia, Vancouver, BC, Canada; ^4^Department of Bioproducts and Biosystems, Aalto University, Espoo, Finland

**Keywords:** lignin, small laccase, dye-decoloring peroxidase, wood, ABTS, mediator, ToF-SIMS

## Abstract

The relative ability of the small laccase (sLac) and dye-decoloring peroxidase (DyP2) from *Amycolatopsis* sp. 75iv2 to transform a variety of lignins was investigated using time-of-flight secondary ion mass spectrometry (ToF-SIMS). The enzymes modified organosolv hardwood lignin to different extents even in the absence of an added mediator. More particularly, sLac decreased the lignin modification metric S (S-lignin)/Ar (total aromatics) by 58% over 16h, while DyP2 lowered this ratio by 31% in the absence of exogenous H_2_O_2_. When used on their own, both sLac and DyP2 also modified native lignin present in aspen wood powder, albeit to lesser extents than in the organosolv lignin. The addition of ABTS for sLac and Mn^2+^ as well as H_2_O_2_ for DyP2 led to increased lignin modification in aspen wood powder as reflected by a decrease in the G/Ar metric by up to a further 13%. This highlights the importance of exogenous mediators for transforming lignin within its native matrix. Furthermore, the addition of ABTS reduced the selectivity of sLac for S-lignin over G-lignin, indicating that the mediator also altered the product profiles. Finally, when sLac was included in reactions containing DyP2, in part to generate H_2_O_2_ *in situ*, the relative abundance of lignin products differed from individual enzymatic treatments. Overall, these results identify possible routes to tuning lignin modification or delignification through choice of enzyme and mediator. Moreover, the current study expands the application of ToF-SIMS to evaluating enzyme action on technical lignins, which can accelerate the discovery and engineering of industrially relevant enzymes for lignin valorization.

## Introduction

Lignin is an extensively methoxylated aromatic heteropolymer that occurs as a structural component of plant cell walls. The main building blocks of lignin are *p*-coumaryl, coniferyl, and sinapyl alcohols (Supplementary Figure S1), which polymerize to create *p*-hydroxyphenyl (H), guaiacyl (G), and syringyl (S) lignin structures, respectively, linked by C-O and C-C bonds (Vanholme et al., 2019). The relative abundance of these structures in lignin depends on the plant tissue and botanical source (Ralph et al., 2019). For example, lignin from softwood (gymnosperms) is composed mainly of G lignin, whereas that from hardwood (angiosperms) is composed mainly of G-S lignin (Ralph et al., 2019; Vanholme et al., 2019). The higher S-lignin content of hardwood lignin means that there are fewer intersubunit C-C bonds.

Oxidative enzymes, including laccases (EC 1.10.3.2.) and various peroxidases (EC. 1.11.1.-), have been studied for their ability to transform lignins (Eggert et al., 1997; Wong, 2009; Lundell et al., 2010; Munk et al., 2015; Biko et al., 2020; Weiss et al., 2020). Laccases are multicopper-dependent enzymes that catalyze the one-electron oxidation of a broad range of compounds, including polyphenols, methoxy-substituted phenols, and aromatic diamines, with concomitant reduction of molecular oxygen to water (Baldrian, 2006; Mate and Alcalde, 2017; Janusz et al., 2020). Laccase-mediator systems enable the oxidation of comparatively high molecular weight substrates like lignin *via* direct electron or hydrogen atom transfer, depending on the mediator (Calcaterra et al., 2008; Jones and Solomon, 2015; Zucca et al., 2016). Heme-dependent peroxidases, including manganese peroxidases (EC.1.11.1.13), lignin peroxidases (EC 1.11.1.14), versatile peroxidases (EC 1.11.1.16), and dye-decolorizing peroxidases (DyP, EC 1.11.1.19), utilize hydrogen peroxide (H_2_O_2_) instead of molecular oxygen as the primary oxidant (Pollegioni et al., 2015; Martinez et al., 2017). Of these peroxidases, DyPs were initially characterized for their ability to decolorize various industrial dyes, but are now understood to act on a variety of substrates, including isolated and embedded lignins (Colpa et al., 2014; Catucci et al., 2020).

Microbial genome sequencing underscores the prevalence of predicted small laccases (sLac; Machczynski et al., 2004; Wu et al., 2020) and DyPs in bacteria (Brown et al., 2012; Singh et al., 2012; Colpa et al., 2014). For example, the biomass-degrading soil bacterium *Amycolatopsis* sp. 75iv2 ATCC 39116 (formerly *Streptomyces setonii* and *S. griseus* 75vi2; Brown et al., 2011) encodes sLac and DyP2, where DyP2 also acts as a manganese peroxidase, using H_2_O_2_ to oxidize Mn^2+^ to Mn^3+^ (Brown et al., 2011, 2012). Structures of sLac and DyP2 have been solved (Brown et al., 2012; Majumdar et al., 2014), and both enzymes were previously shown to oxidize 2,2'-azino-bis(3-ethylbenzothiazoline-6-sulfonic acid) (ABTS) as well as a wide range of monoaryls and model lignin compounds (Brown et al., 2012; Singh et al., 2017). The ability of sLac to transform lignin has also been evaluated using organosolv lignin and ball-milled birch wood (Perna et al., 2020). In their study of laccases, Perna et al. (2020) showed that laccase-mediated oxidation of lignin led to the formation of H_2_O_2_, which is a co-substrate of other oxidative enzymes involved in lignocellulose deconstruction. For example, Perna et al. (2020) demonstrated the activation of lytic polysaccharide monooxygenases (LPMOs) by laccase-generated H_2_O_2_. Given their occurrence in the same bacterium, the potential of H_2_O_2_ from sLac to boost DyP2 activity on lignin warrants investigation.

A number of methodologies have been used to characterize the action of laccases and peroxidases on technical and native lignins. For example, two-dimensional nuclear magnetic resonance spectroscopy (e.g., 2D-HSQC-NMR) and pyrolysis gas chromatography - mass spectrometry (Py-GC–MS) have been used to establish that laccase acts on lignin in the absence of exogenous mediators (Rico et al., 2014; Singh et al., 2017; van Erven et al., 2020). Surface compositional analysis methods, such as Time-of-Flight Secondary Ion Mass Spectrometry (ToF-SIMS), have also been used to analyze enzyme action on native lignin present in lignocellulose (Saito et al., 2005; Goacher et al., 2011, 2014). Importantly, direct analysis of enzyme-treated biomass by ToF-SIMS facilitated the development of 96-well-based enzyme screens using industrially relevant substrates (Goacher et al., 2013, 2018).

**Figure 1:**
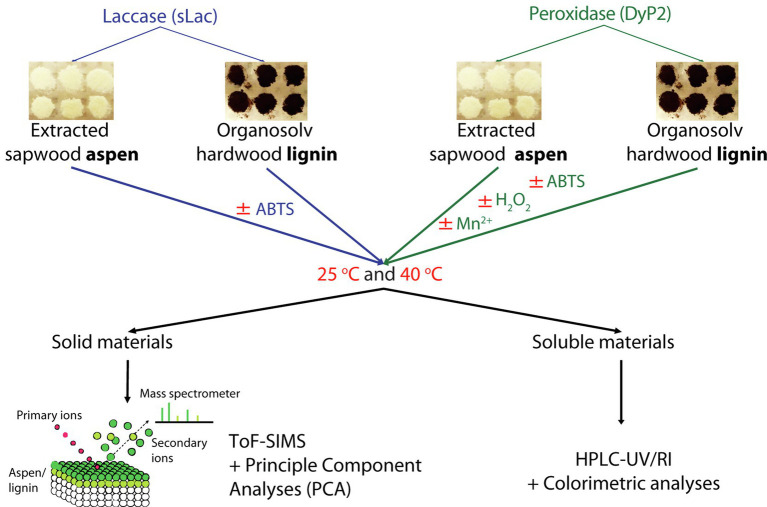
Flowchart for analyzing activity of sLac and DyP2 on organosolv lignin and wood powder. Both enzymes were incubated at 25 and 40°C with and without co-substrates/mediator. The reactions were filtered through a 1.2μm PVDF membrane: the filtrates were then analyzed by HPLC-UV/RI and spectrophotometry while the remaining solid samples were analyzed by ToF-SIMS.

In this study, ToF-SIMS was used to study the ability of sLac and DyP2 from *Amycolatopsis* sp. 75iv2 to transform native lignin present in aspen wood powder and organosolv lignin isolated from hardwood (Figure 1). The impact of exogenous mediators was also evaluated. While ToF-SIMS has been used to characterize a commercial fungal laccase (Goacher et al., 2012), a lignin peroxidase and manganese peroxidase (MacDonald et al., 2016), and recombinant bacterial laccases (Goacher et al., 2018) on ground wood samples, the current study extends the application of ToF-SIMS for enzyme screening directly on technical lignin. This investigation revealed that both sLac and DyP2 transform native and organosolv lignins in the absence of exogenous mediators, and generate product profiles that depend on both the enzyme type and presence of mediators. By demonstrating the capacity of ToF-SIMS to compare different lignin-active enzymes on technical lignins, our study advances the application of this method for the discovery and engineering of new industrial biocatalysts.

## Materials and Methods

### Wood and Lignin Preparation

Sapwood from trembling aspen (*Populus tremuloides*) was ball-milled using a Wiley mill (Thomas Scientific, NJ, USA). Aspen wood powders were sifted through a USA standard 100-mesh size sieve, with an opening diameter of 150μm (Goacher et al., 2012). The wood powders were then sequentially extracted in a Soxhlet apparatus with ethanol, toluene, and water following ASTM Standard D1105-96, to remove small extractives, which can overlap with lignin peaks and alter ToF-SIMS peak ratios (Goacher et al., 2013). Organosolv hardwood lignin was obtained from Suzano Canada Inc. (previously Lignol Innovations, Vancouver, Canada); its quality and purity were previously confirmed (Arefmanesh et al., 2020).

Existing H_2_O_2_ concentration in wood and isolated lignin samples was measured using the Amplex® Red/peroxidase assay kit (Thermo Fisher Scientifics, USA). Briefly, 3mg of each solid sample were mixed with 50μM Amplex® Red agent and 0.1U/ml horseradish peroxidase at 37°C in MilliQ water. As a control, 20mM potassium iodide was added to decompose existing H_2_O_2_. The reaction was vacuum-filtered using a 1.2μm PVDF membrane, and the flow-through was collected into 96-well black microplates for measuring fluorescence intensity at an excitation wavelength of 530nm and an emission wavelength of 590nm.

### Protein Purification and Initial Assays

*Amycolatopsis* sp. 75iv2 ATCC 39116 laccase (sLac, Genbank accession: WP_020416648) was heterologously produced in *Escherichia coli*, as described previously (Singh et al., 2017). *Amycolatopsis* sp. 75iv2 ATCC 39116 dye-decoloring peroxidase (DyP2, Genbank accession: WP_020421762) was produced with an N-terminal His_10_-tag containing a linker with a Tobacco Etch Virus protease site in *E. coli* BL21(DE3), as described (Brown et al., 2012). Purified proteins were flash frozen in liquid nitrogen and stored at −80°C. Protein purity was evaluated using SDS-PAGE (Supplementary Figure S2). The electrostatic properties of each protein were evaluated using the Adaptive Poisson-Boltzmann Solver.^1^

Enzyme activities were measured at pH 4.5, which is within the pH range of both enzymes (Brown et al., 2012; Majumdar et al., 2014) and optimum pH of DyP2 (Brown et al., 2012). Sodium malonate was used as the buffer since DyP2 requires natural Mn^3+^ chelators to act as a manganese peroxidase (Brown et al., 2012). Specifically, enzyme activity on 0.1mM ABTS was assayed in 50mM sodium malonate pH 4.5 in 96-well microplates; in case of DyP2, 0.1mM H_2_O_2_ was also added. The reactions were measured continuously for 50min at an absorbance of 420nm at 25°C using an Infinite 200 plate reader (Tecan Trading AG, Switzerland).

### Enzyme Treatment of Wood Powder and Organosolv Lignin

Aspen wood powder and organosolv hardwood lignin (3mg) were weighed in triplicates to 96-well filter plates fitted with a 1.2-μm PVDF membrane (Millipore, USA). The total reaction volume was 200μl with the final concentrations: 0.2mg/ml enzyme, 0.1mM ABTS, 0.1mM MnSO_4_, 0.1mM H_2_O_2_, and 50mM sodium malonate pH 4.5 (Figure 1). Tween 80 was not used as it appeared to solubilize the organosolv lignin, leading to lignin loss during the washing steps. sLac was denatured by boiling at 100°C for 10min and used as a negative control. The reactions were incubated for 16h at 600rpm on Eppendorf thermomixers; reactions were performed at 25°C unless otherwise specified. After incubation, the flow-through was collected by filtration through the 1.2-μm PVDF membrane using a Tecan liquid handler equipped with vacuum filtration (Tecan Trading AG, Switzerland). Reaction retentates were washed 10 times with MilliQ water to remove ABTS that might interfere with lignin modification metrics (Goacher et al., 2018). The powders were then air-dried overnight and attached to glass slides by double-sided tape for ToF-SIMS analysis.

### Soluble Product Analysis

The flow-through from each reaction was collected into a Quartz 96-well microplate and the absorbance of each well was scanned from 200nm to 800nm using an Infinite 200 plate reader (Tecan Trading AG, Switzerland). The filtrates were also analyzed by High Performance Liquid Chromatography (HPLC). In this case, each sample (12.5μl) was injected to a Dionex Ultimate 3000 system equipped with an Aminex HPX-87H column (300mm×7.8mm, Bio-Rad cat.no. 125–0140). H_2_SO_4_ (5mM) was used as the eluent at a flow rate of 0.6ml/min, and eluted products were detected by an UV detector (DAD-3000) at wavelengths of 254nm and 280nm, as well as by a Shodex RI-101 differential refractive index detector. Each run was 40min at 50°C. Chromatograms were analyzed using Chromeleon 7.2 (Dionex, USA).

### ToF-SIMS Data Acquisition and Analysis

ToF-SIMS spectra were acquired in a positive mode using a ToF-SIMS V instrument (IONTOF GmbH, Münster, Germany). All samples were analyzed with 50keV Bi_3_^2+^ high current bunched primary ions with a pulsed current of approximately 0.3 pA. Six different spectra were acquired for each sample. The primary ions were randomly rastered over 500μm×500μm area with 128×128 pixels. Charge neutralization was performed using 20eV electron flooding. Ion doses were kept below 1×10^11^ ions/cm^2^ to prevent sample damage. The mass spectra were calibrated to CH_3_^+^, C_2_H_3_^+^, C_3_H_5_^+^, and C_4_H_7_^+^ ions using SurfaceLab v6 (IONTOF GmbH, Muenster, Germany). Principal component analysis (PCA) was performed in MATLAB R2014a (The Mathworks Inc., USA) with PLS Toolbox v7 (Eigenvector Research Inc., USA) and in R. Clustering heatmap analysis with the Euclidean distance and the Ward clustering algorithm was carried out in R.^2^

The G-lignin modification metric (G/Ar) is calculated by summating the intensity of peaks corresponding to intact methoxylated G-lignin (peaks at *m/z* 137 and 151; Saito et al., 2005), and dividing that value by the combined intensity of peaks for nonfunctionalized aromatic rings (Ar; peaks at *m/z* 77 and 91; Saito et al., 2005). Similarly, the S-lignin modification metric (S/Ar) is calculated by summating the intensity of peaks corresponding to intact methoxylated S-lignin (peaks at *m/z* 167 and 181; Saito et al., 2005), divided by the corresponding value for Ar. The lignin degradation metric is L/(L+PS), where L and PS are the sums of peaks determined to characterize lignin and polysaccharides, respectively (Goacher et al., 2012, 2018; MacDonald et al., 2016).

Statistical analyses were conducted using one-way Analysis of Variance followed by Tukey’s multiple comparison test in PRISM v5 (GraphPad Software Inc., USA).

## Results and Discussion

### sLac and DyP2 Modified Organosolv Lignin in the Absence of Added Mediators

Enzymatic modification of lignin is typically evaluated in the presence of added mediators, such as ABTS (Christopher et al., 2014; Hilgers et al., 2018). However, several recent studies have established that these enzymes can act on lignins in the absence of such mediators (Rico et al., 2014; Choolaei et al., 2020; Perna et al., 2020). Accordingly, we tested the ability of sLac and DyP2, two bacterial enzymes, to modify organosolv hardwood lignin and aspen wood powder in the presence and absence of ABTS or Mn^2+^. In addition, we investigated the lignin-modifying capability of DyP2 in the absence of exogenously added H_2_O_2_. Lignin modification metrics G/Ar and S/Ar were calculated from ToF-SIMS spectra of the residual sample, where G/Ar corresponds to the combined intensity of peaks assigned to intact methoxylated G-lignin (G) over the combined intensity of peaks for nonfunctionalized aromatic rings (Ar), and S/Ar corresponds to the combined intensity of peaks assigned to intact methoxylated S-lignin (S) over Ar (Goacher et al., 2012, 2018; MacDonald et al., 2016).

**Figure 2:**
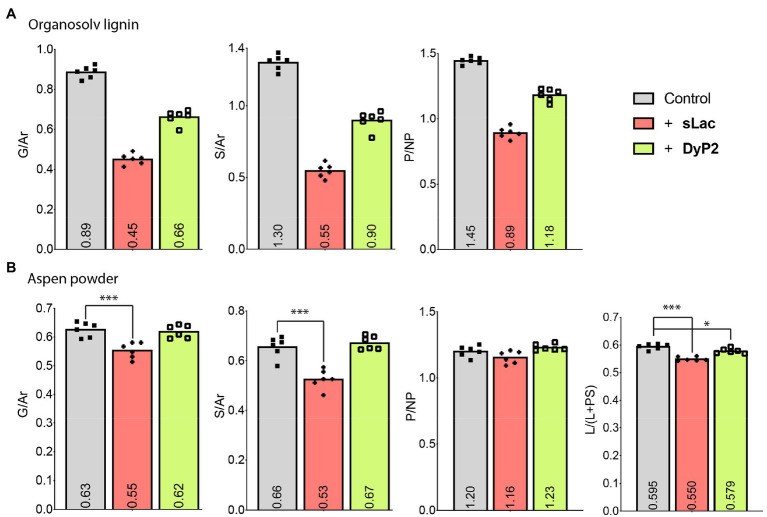
The effect of sLac and DyP2 alone on organosolv lignin **(A)** and lignin in aspen wood powder **(B)**. G, S, Ar, L, PS, P, and NP are the combined intensity of peaks corresponding to intact methoxylated G-lignin, intact methoxylated S-lignin, nonfunctionalized aromatic rings, lignins, polysaccharides, phenolic structures, and non-phenolic structures, correspondingly. Six ToF-SIMS spectra (*n*=6) of each sample were acquired, shown as scattering dots, and their averages were reported as bar charts as well as numbers inside columns. ANOVA analysis with Tukey’s post-test for **(B)**: ^*^ and ^***^ mean *p*=0.02 and *p*<0.0001, respectively.

Both sLac and DyP2 modified the G-lignin and S-lignin components of organosolv lignin in a dose-dependent manner in the absence of an added mediator (Supplementary Figure S3). More specifically, sLac reduced S/Ar by up to 58% while DyP2 lowered this ratio by 31% (Figure 2). The decreased G/Ar and S/Ar ratios are consistent with the oxidation of the methoxy and phenol groups of the lignin’s aromatic rings. A phenolic modification metric (P/NP) was defined here as the ratio of combined peak intensities for phenolics (P; lignin peaks with *m/z* values equal or greater than 94) and combined peak intensities for non-phenolics (NP; lignin peaks at *m/z* lower than 94). Consistent with laccases showing preference towards phenolics (Christopher et al., 2014), sLac reduced the P/NP ratio of organosolv lignin by 39% whereas DyP2 reduced the P/NP ratio by 19% (Figure 2).

Reaction filtrates recovered following the treatment of organosolv lignin with sLac or DyP2 showed higher UV absorbance compared to control reactions lacking enzymes (Supplementary Figure S4A). This result suggests both enzymes were able to break down the organosolv lignin in the absence of an added mediator, releasing soluble products. To study whether these enzymes could also directly act on native lignin present in a lignocellulose matrix, the reactivity of sLac and DyP2 with aspen wood powder was then investigated.

### sLac and DyP2 Modified Native Lignin in Wood in the Absence of Added Mediators

In the absence of an added mediator (e.g., ABTS), sLac transformed G-lignin and S-lignin components of native lignin present in aspen wood powder (Figure 2). Similar to its action on organosolv lignin, sLac preferentially modified S-lignin over G-lignin (20 and 13%, respectively) in the native lignin substrate (Figure 2). This result is in agreement with earlier 2D-NMR analysis of sLac action on lignin, which showed preferential oxidation of S-lignin in steam-pretreated poplar (Singh et al., 2017). Preferential transformation of S-lignin is probably because S-lignin has fewer aryl-aryl bonds and a lower redox potential compared to G-lignin (Martinez et al., 2001). The evidence of delignification by sLac could be seen when calculating the lignin degradation metric L/(L+PS) (Figure 2), where the intensity of lignin peaks (L) is summed and divided by (L) plus the total intensity of polysaccharide peaks (PS) (Goacher et al., 2012). Although the decrease in L/(L+PS) by sLac was low (8%), delignification of the wood sample by sLac alone was supported by the appearance of an UV-absorbing peak in reaction supernatants (Supplementary Figure S4B).

**Figure 3:**
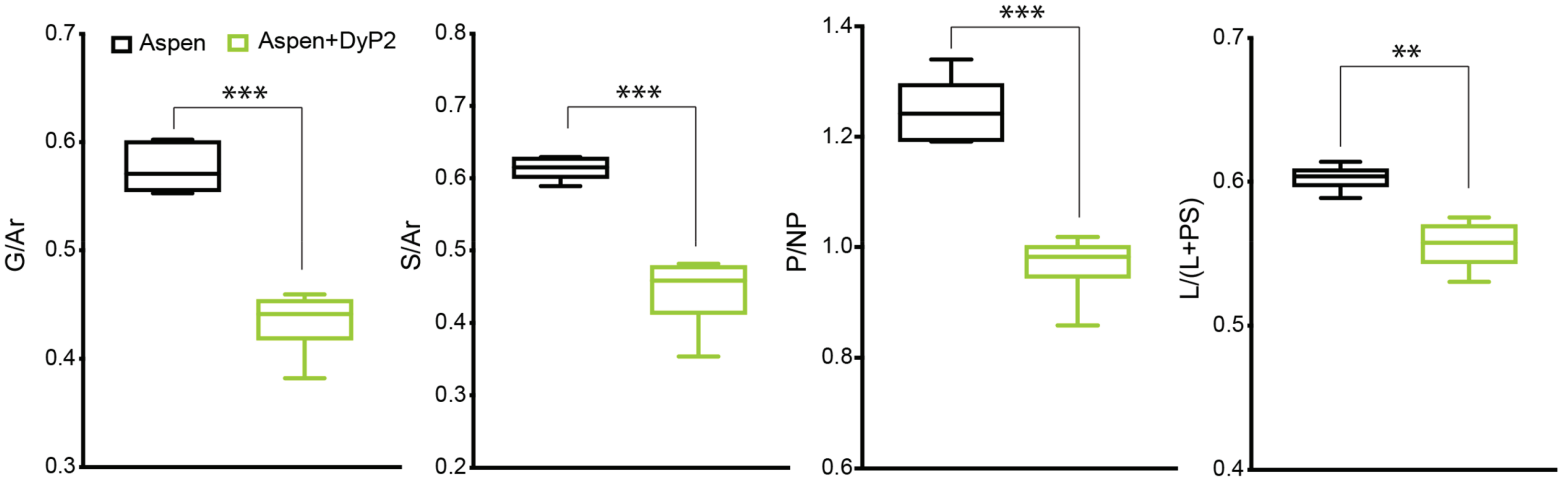
Modification of lignin in aspen wood powder by DyP2. Reactions were performed at 40°C. Lignin modification metrics, G/Ar, S/Ar, P/NP, and L/(L+PS) ratios are presented, from left to right. Data are based on six ToF-SIMS spectra of each treatment (*n*=6); ANOVA analysis with Tukey’s post-test: ^**^p<0.005; ^***^p<0.0001, compared to the no-enzyme control.

DyP2 did not substantially change the lignin composition of the wood powder at the standard conditions used in this study, i.e., at 25°C (Figure 2). However, when increasing the reaction temperature to 40°C, which approaches the temperature used to induce lignin transformation by *Amycolatopsis* sp. 75iv2 (Brown et al., 2011), all four lignin modification metrics (i.e., G/Ar, S/Ar, P/NP, and L/(L+PS) ratios) decreased by up to 24% (Figure 3).

### Organosolv Lignin and Wood Powder Contain Co-substrates and Mediators

The results above showed that both sLac and DyP2 modified lignin without the addition of mediators nor co-substrate H_2_O_2_ in the case of DyP2; therefore, these enzymes either acted directly on lignin, or used unidentified mediators in the organosolv lignin and wood powder samples. For example, sLac transforms syringate, vanillate, protocatechuate, and syringaldehyde (Singh et al., 2017), which might already exist in extracted lignin and wood. On the other hand, lignin modification by DyP2 (Figure 2) might be driven by its co-substrate H_2_O_2_ intrinsic to the samples. Measurements of pre-existing H_2_O_2_ in the isolated lignin and aspen wood powder samples used in this study showed that these samples contained nearly 5μM H_2_O_2_ (Supplementary Figure S5). Similarly, H_2_O_2_ was recently measured in organosolv lignin (from Sigma) and birch wood at 5.2μM and 4.4μM, respectively (Perna et al., 2020). Pre-existing H_2_O_2_ in the organosolv lignin and wood powder samples could promote the peroxidase cycle catalyzed by DyP2. For instance, in the presence of H_2_O_2_ and ABTS, DyP2 breaks down the lignin model dimer guaiacylglycerol-β-guaiacol ether (Brown et al., 2011). Alternatively, DyP2 might use pre-existing manganese as a natural mediator for lignin modification, as DyP2 was previously shown to carry out an oxidative decarboxylation reaction in the presence of just O_2_ and Mn^2+^ (Brown et al., 2012). Manganese is likely present in the wood substrate (Krutul et al., 2017) and organosolv lignin used herein, as the addition of Mn^2+^ to lignin did not create a distinguishable cluster from lignin alone in the PCA analysis of ToF-SIMS spectra (Supplementary Figure S6).

### The Addition of Mediators Altered Lignin Modification by sLac and DyP2

Even though both sLac and DyP2 modified lignins in the absence of added mediators, the extent of lignin modification was less in aspen wood powder compared to organosolv lignin (Figure 2). Therefore, the potential to increase the enzymatic modification of lignin through addition of exogenous mediators was investigated. Since both sLac and DyP2 efficiently oxidize ABTS (Brown et al., 2012; Singh et al., 2017), ABTS was chosen as the mediator to facilitate direct comparisons of the enzymes (Supplementary Figure S7).

**Figure 4:**
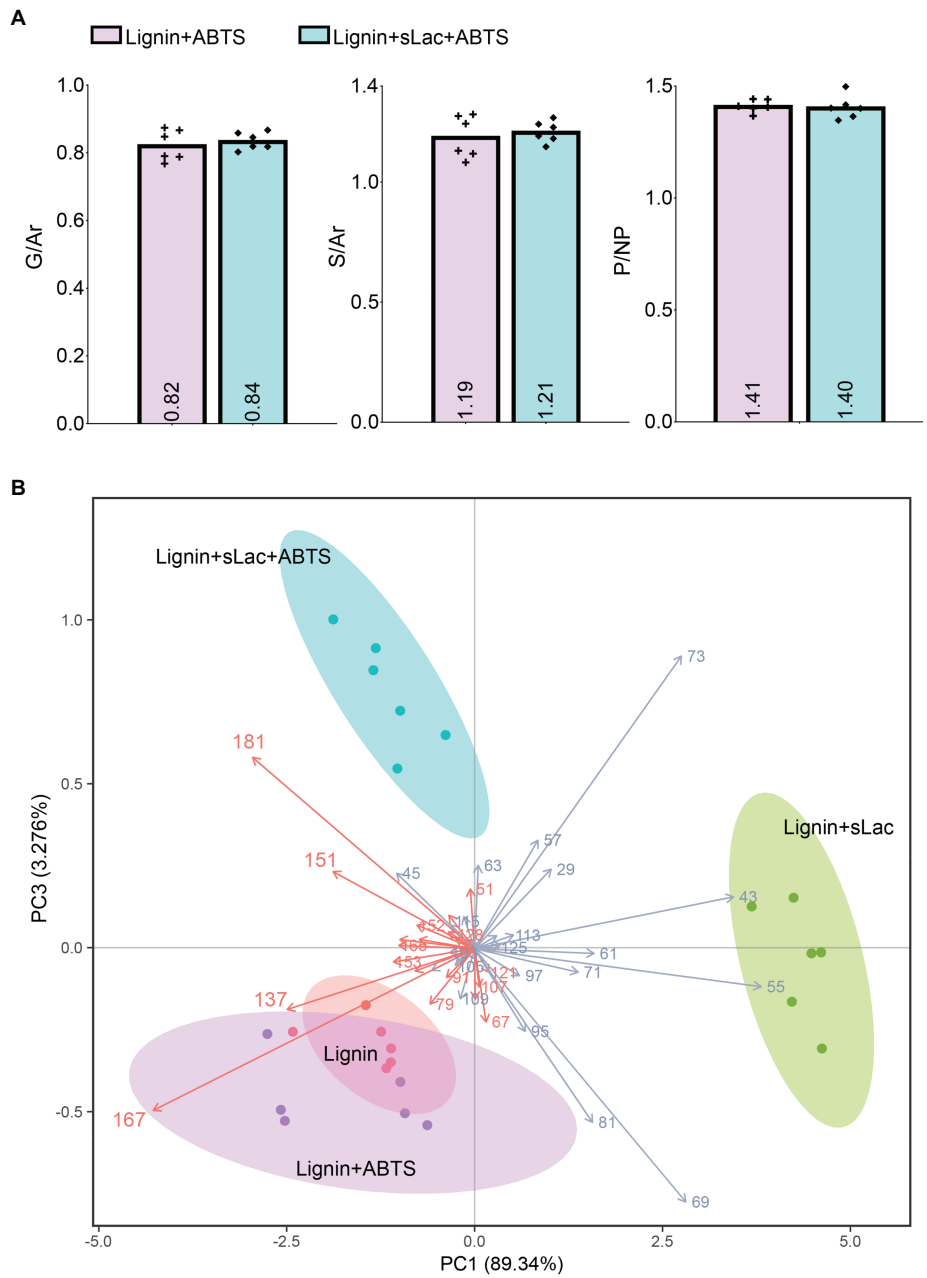
Effect of ABTS on sLac activity on organosolv lignin. **(A)** G/Ar, S/Ar, and P/NP ratios: Data are shown as dots and their averages are reported as bar charts as well as numbers inside columns. **(B)** PCA analysis: The numbers on the top of arrows indicate *m/z* values, those annotated as lignin are highlighted in red. The arrows demonstrate the influence of *m/z* values in sample clustering. Ellipses represent 95% confidence of sample distribution, colored dots are ToF-SIMS acquisition data (*n*=6).

**Figure 5:**
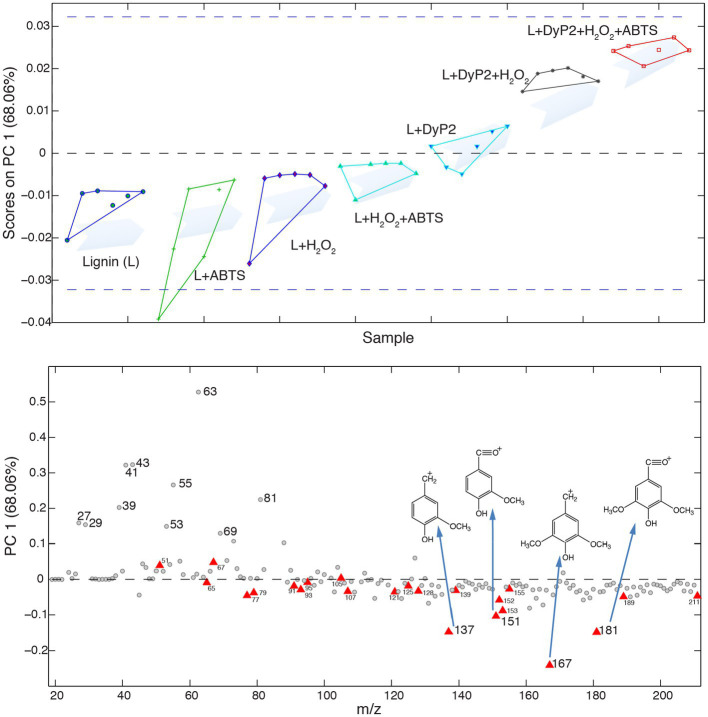
ABTS enhanced lignin modification of DyP2 on organosolv lignin. PC1 score (top) and PC1 loading (bottom) of six lignin treatments (*n*=6). Boundaries of ToF-SIMS acquisition data of each treatment are shown by lines. Lignin peaks are highlighted as red triangles, and the structures of lignin peaks that contributed most to clustering are inserted in the PC1 loading chart and highlighted by blue arrows.

**Figure 6:**
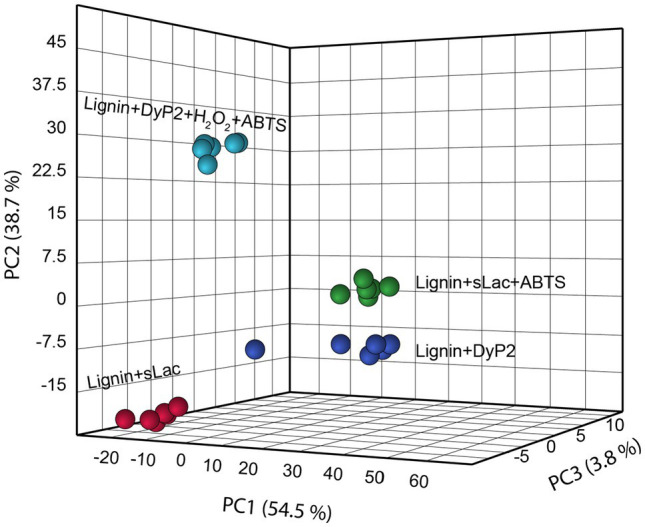
DyP2 and sLac created different PCA clusters in organosolv lignin in the absence or presence of ABTS. Six ToF-SIMS data (*n*=6) of each treatment were acquired as shown as colored balls.

The addition of ABTS did not substantially change the extent to which organosolv lignin was modified by sLac (Figure 4A). However, closer inspection of the corresponding ToF-SIMS spectra showed that sLac treatments with ABTS led to products with higher peak intensity at *m/z* 151 and 181, and lower peak intensity at *m/z* 137 and 167, compared to treatments with sLac alone (Figure 4B). Corresponding peak assignments (Figure 5) would indicate an attack at the C_α_ positions of both G and S-lignin units in the presence of ABTS. The addition of ABTS to reactions comprising sLac and organosolv lignin also increased the release of UV–Vis absorbing products from the organosolv lignin, where absorbances at 420nm and 500nm increased by 130 and 344%, respectively (Supplementary Figure S8). Similarly, the addition of ABTS to reactions comprising DyP2, organosolv lignin and H_2_O_2_ led to a product profile characterized by decreased peak intensity at *m/z* 137 and 167 compared to corresponding reactions that lacked ABTS (Figure 5). Thus, both enzyme and added mediator impact the profile of products that can be generated from organosolv lignin (Figure 6).

The addition of ABTS to aspen wood samples further increased lignin modification by sLac by up to 13% (Table 1). At the same time, the addition of ABTS appeared to reduce sLac selectivity towards S-lignin structures (Table 1). The preferential transformation of S-lignin by sLac is consistent with earlier screens of bacterial and fungal laccases on different hardwood powders (Goacher et al., 2018), as well as the previously reported impacts of laccase mediators on product profiles from softwood kraft lignin (Wang et al., 2018). Neither the addition of ABTS nor H_2_O_2_ significantly increased the modification of native lignin by DyP2. Instead, adding both Mn^2+^ and H_2_O_2_ to DyP2 reactions increased the modification of native lignin in aspen wood powder by approximately 10% (Table 1), consistent with previous studies showing DyP2 as a manganese peroxidase and Mn^2+^-dependent oxidase (Brown et al., 2012). Although not essential to enzyme action, the clear benefit of exogenous mediators to both sLac and DyP2 action on wood underscores the recognized challenge of substrate accessibility when targeting native lignins present in wood and other lignocellulosic materials. Moreover, mediators with high redox potentials would extend the product profile to include non-phenolic moieties in lignin, particularly in natural lignin that are inaccessible by the enzymes.

**Table 1:** The effect of mediator on the ability of sLac and DyP2 to modify native lignin present in aspen wood powder.^a^

	L/(L+PS)	P/NP	G/Ar	S/Ar
H_2_O_2_	0.590±0.006	1.23±0.03	0.64±0.02	0.68±0.02
Mn^2+^	0.599±0.004	1.25±0.05	0.64±0.04	0.69±0.03
ABTS	0.587±0.012	1.24±0.04	0.66±0.02	0.69±0.04
ABTS+H_2_O_2_	0.588±0.015	1.29±0.04	0.68±0.01	0.73±0.04
sLac+ABTS	0.580±0.011	1.12±0.04^***^	0.48±0.02^***^	0.48±0.02^***^
DyP2+H_2_O_2_	0.583±0.006	1.23±0.03	0.61±0.02	0.67±0.02
DyP2+H_2_O_2_+ABTS	0.582±0.010	1.18±0.05	0.59±0.02	0.63±0.04
DyP2+H_2_O_2_+Mn^2+^	0.566±0.009^**^	1.16±0.04	0.55±0.03^***^	0.62±0.03^*^

a Lignin modification metrics are indicated and defined in the main text. All reactions contained powdered aspen. Reported values are based on six ToF-SIMS spectra (*n*=6). ANOVA analysis with Tukey’s post-test: *p<0.05; **p<0.005; ***p<0.0005 compared to the no-enzyme controls, including aspen wood powder alone. Refer to Figure 2 for the conditions of aspen, aspen+sLac, and aspen+DyP2.

### Evaluating the Impact of sLac and DyP2 Co-treatments on Lignin Product Profiles

**Figure 7:**
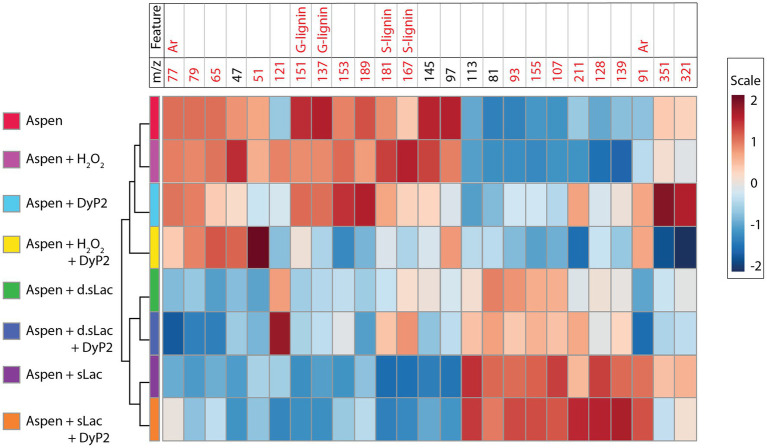
Hierarchical clustering heatmap of aspen wood powder treated with different enzymes. Samples (average of six acquisition data) are in columns. sLac and DyP2, 0.2mg/ml each, were added to individual or mixture treatments. Heat-denatured sLac (d.sLac) was included as a control of sLac. The top 25 ToF-SIMS peaks, ranked by *t*-test/ANOVA, are shown in columns; the *m/z* values for lignin are written in red while *m/z* values for polysaccharides are written in black. Feature peaks for G-lignin, S-lignin, and aromatic rings (Ar) (Saito et al., 2005) are labeled.

Although H_2_O_2_ occurs in organosolv lignins and aspen wood powder, the oxidation of lignin by laccases, including sLac, can also generate H_2_O_2_ (Perna et al., 2020). It is thus conceivable that sLac could boost DyP2 action. The action of one enzyme on lignin might also alter the other’s accessibility to the substrate. To investigate these possibilities, aspen wood powder was simultaneously treated with sLac and DyP2 in the absence of an added mediator (Figure 7). The product profile resulting from the combined sLac and DyP2 treatment was not significantly different from that of sLac alone, based on PCA analysis of ToF-SIMS spectra. This could be because the reduction potential of sLac is expected to be higher than DyP2 given that sLac alone modified a broader range of structures to higher extent within lignin compared to DyP2 (Figures 2, 7). Alternatively, differences in molecular weight and surface charge of each enzyme could influence the co-location and coordinated action of sLac and DyP2 on native lignin in the aspen wood powder. Briefly, sLac is a trimer of 31kDa subunits (Majumdar et al., 2014), whereas DyP2 is an oligomer with n of 4 to 6 of 50kDa subunits (Brown et al., 2012), and each enzyme displays a distinct net surface charge (Supplementary Figure S9). Even though the hierarchical clustering of product spectra grouped samples treated with both sLac and DyP2 with samples treated with sLac alone, slight differences in the relative abundance of several lignin peaks was observed (Figure 7). For example, the addition of sLac to DyP2 lowered the peak intensity at *m/z* 121, which is derived from either H-lignin (Goacher et al., 2012) or extractives (Goacher et al., 2013), while increasing the peak intensity at *m/z* 211 attributed to syringyl alcohol (Banoub et al., 2015).

## Conclusion

Our study demonstrates the application of ToF-SIMS to evaluate the actions of a variety of enzymes on diverse lignins. These results extend the application of this technique in enzyme screens that reach beyond the use of model compounds or mediator substrates. The use of ToF-SIMS helps to characterize how DyP2 acts on organosolv lignin and native lignin present in wood powder, and confirmed the ability of sLac and DyP2 to directly modify lignin in the absence of an added mediator and H_2_O_2_. Both enzymes modified organosolv lignin to a greater extent than lignin in aspen wood powder. The nature of the modifications to the lignins, however, varied depending on the choice of enzymes and mediators. For example, sLac alone preferentially modified S-lignin over G-lignin in the absence of exogenous mediators, a preference that was minimized in the presence of ABTS. These results open possibilities to tune to the modification of lignin through choice of enzyme and mediator. Lastly, the addition of sLac to reactions with DyP2 for *in situ* H_2_O_2_ generation did not dramatically impact lignin transformation in aspen wood powder. However, differences in relative abundances of specific lignin products were detected, highlighting potential synergy between lignin-active enzymes and the importance of further study of laccase/peroxidase systems to transform lignin.

## Data Availability Statement

The original contributions presented in the study are included in the article/Supplementary Material, further inquiries can be directed to the corresponding author.

## Author Contributions

ERM, TVV, LDE, and RS contributed to the conception and design of the study and manuscript revision. TVV conducted ToF-SIMS, HPLC experiments, and PCA analyses. TVV and ERM drafted the manuscript. All authors contributed to the article and approved the submitted version.

## Funding

This work was supported by the Government of Ontario for the project “Forest FAB: Applied Genomics for Functionalized Fibre and Biochemicals” (grant number ORF-RE-05-005), the Natural Sciences and Engineering Research Council (NSERC) of Canada for the Strategic Network Grant “Industrial Biocatalysis Network,” and Genome Canada for the LSARP project “SYNBIOMICS – Functional genomics and techno-economic models for advanced biopolymer synthesis” (grant number 10405) to ERM as well as NSERC Discovery Grant 171359 to LDE. LDE is the recipient of a Tier 1 Canada Research Chair in Microbial Catabolism and Biocatalysis.

## Conflict of Interest

The authors declare that the research was conducted in the absence of any commercial or financial relationships that could be construed as a potential conflict of interest.

## Publisher’s Note

All claims expressed in this article are solely those of the authors and do not necessarily represent those of their affiliated organizations, or those of the publisher, the editors and the reviewers. Any product that may be evaluated in this article, or claim that may be made by its manufacturer, is not guaranteed or endorsed by the publisher.

## Footnotes

^1^ https://server.poissonboltzmann.org

^2^ www.r-project.org

## Abbreviations

ToF-SIMS: Time-of-flight secondary ion mass spectrometry
sLac: small laccase
DyP: dye-coloring peroxidase
ABTS: 2,2'-azino-bis(3-ethylbenzothiazoline-6-sulfonic acid)
