# Supplementary material for: The Comparative Abilities of a Small Laccase and a Dye-Decoloring Peroxidase From the Same Bacterium to Transform Natural and Technical Lignins
Source: Front Microbiol. 2021 Oct 18;12:723524. doi: 10.3389/fmicb.2021.723524 (PMC8559727; doi:10.3389/fmicb.2021.723524)
Supplement: Supplementary file 1 [file Data_Sheet_1.docx]

**The comparative abilities of a small laccase and a dye-decoloring peroxidase from the same bacterium to transform natural and technical lignins**

Thu V. Vuong^1^, Rahul Singh^2,3^, Lindsay D. Eltis^2^, and Emma R. Master^1,4^

^1^ Department of Chemical Engineering and Applied Chemistry, University of Toronto, Toronto, Canada.

^2^ Department of Microbiology and Immunology, BioProducts Institute, The University of British Columbia, Vancouver, Canada.

^3^ Genome British Columbia, Vancouver, Canada.

^4^ Department of Bioproducts and Biosystems, Aalto University, Espoo, Finland


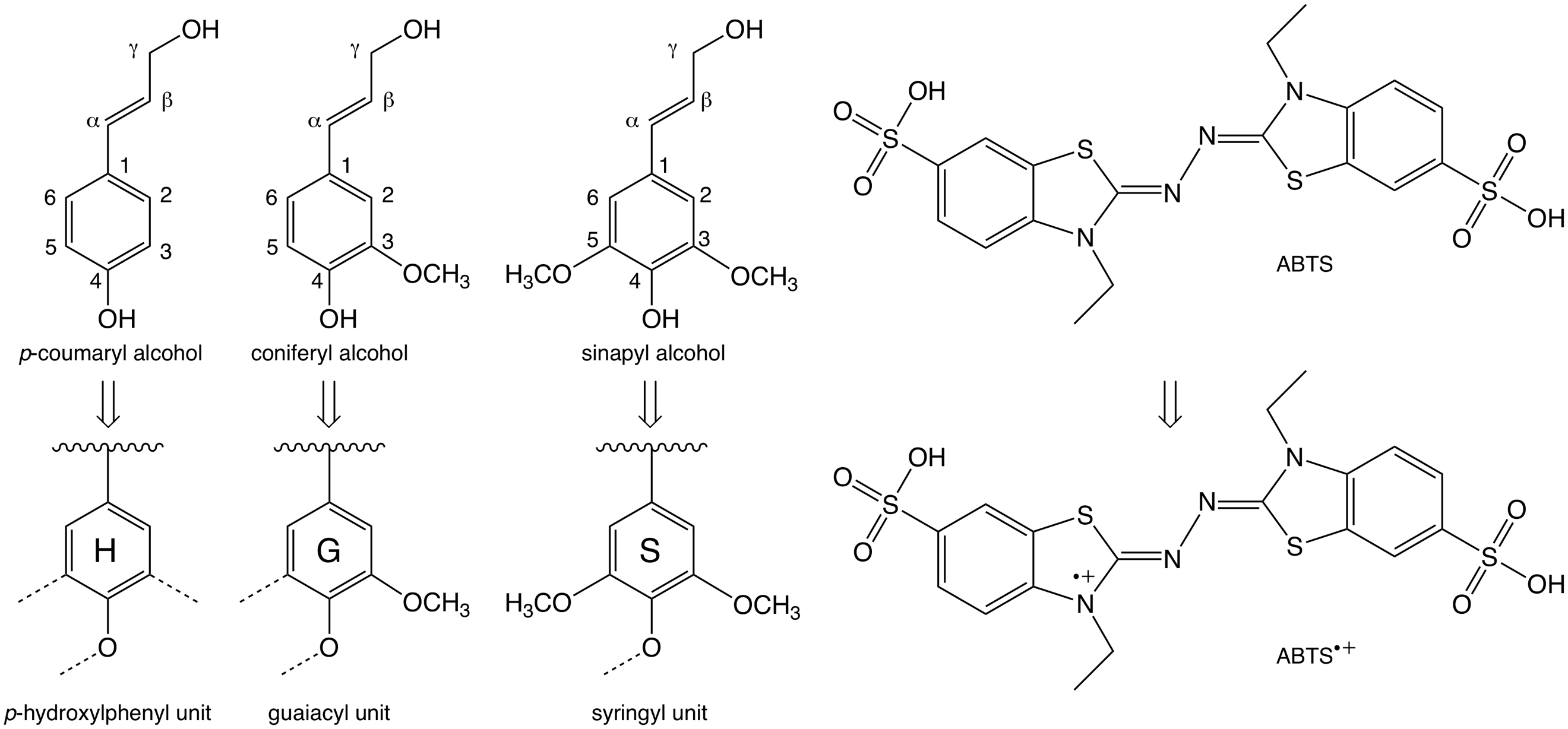


**Figure S1. Structures of key compounds discussed in this work.** Three monolignols (*p*-coumaryl alcohol, coniferyl alcohol and sinapyl alcohol) and their corresponding moieties in lignin; 2,2'-azino-bis(3-ethylbenzothiazoline-6-sulfonic acid (ABTS) and its corresponding radical (ABTS^●+^).


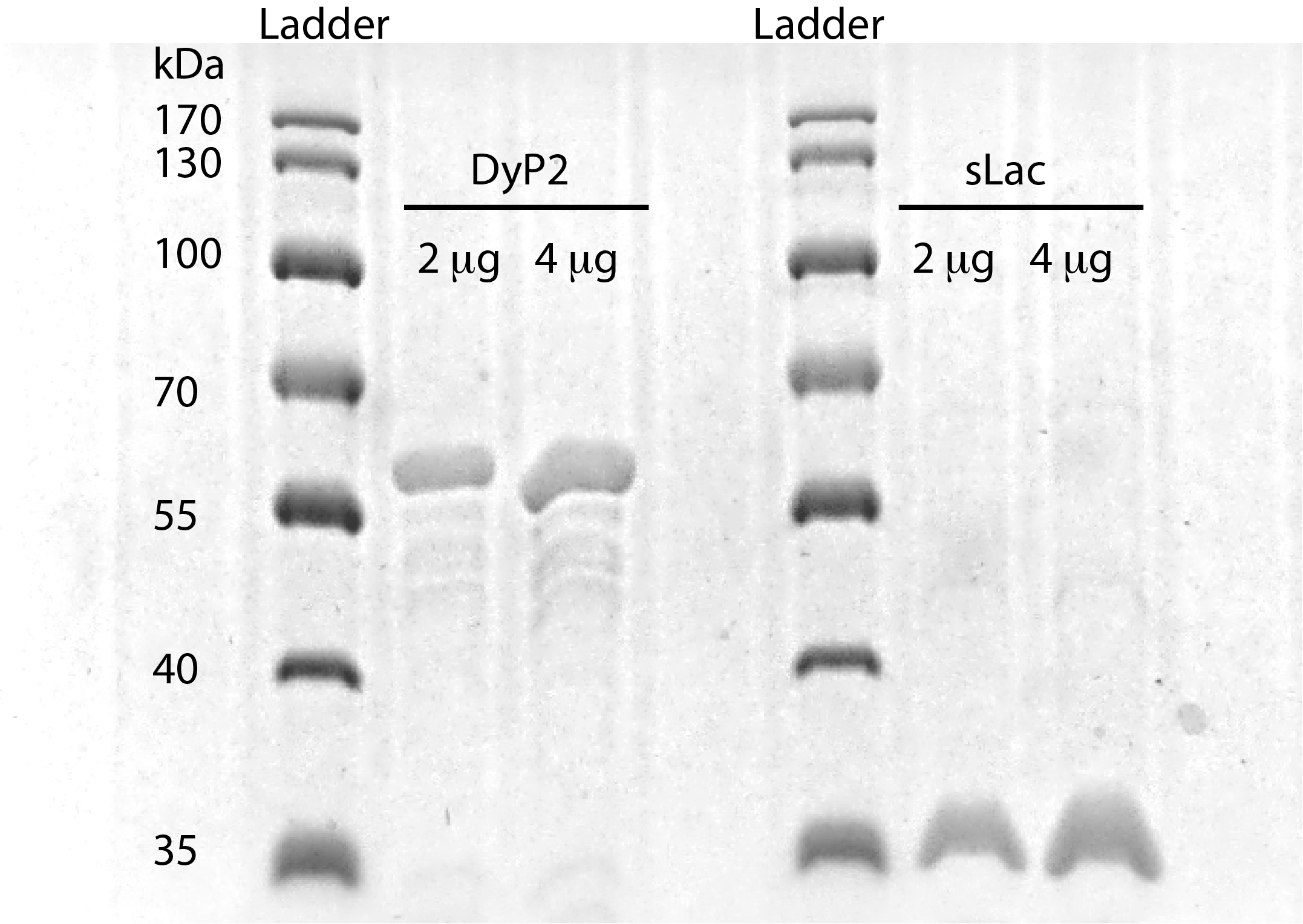


**Figure S2. SDS-PAGE of purified sLac and DyP2.** 2 μg and 4 μg of protein were loaded. The gel was stained with Coomassie Brilliant Blue and imaged using a Bio-Rad ChemiDoc^TM^ XRS+ with Image Lab^TM^ software.

**Figure S3. Dose-dependence of sLac (A) and DyP2 (B) on organosolv lignin.** Enzyme concentrations were 0, 0.05, 0.1, 0.2 and 0.5 μg/μL; the loading of PC1 scores are lignin-, polysaccharide-related peaks. Boundaries of ToF-SIMS acquisition data (n=6) of each treatment were shown by lines.


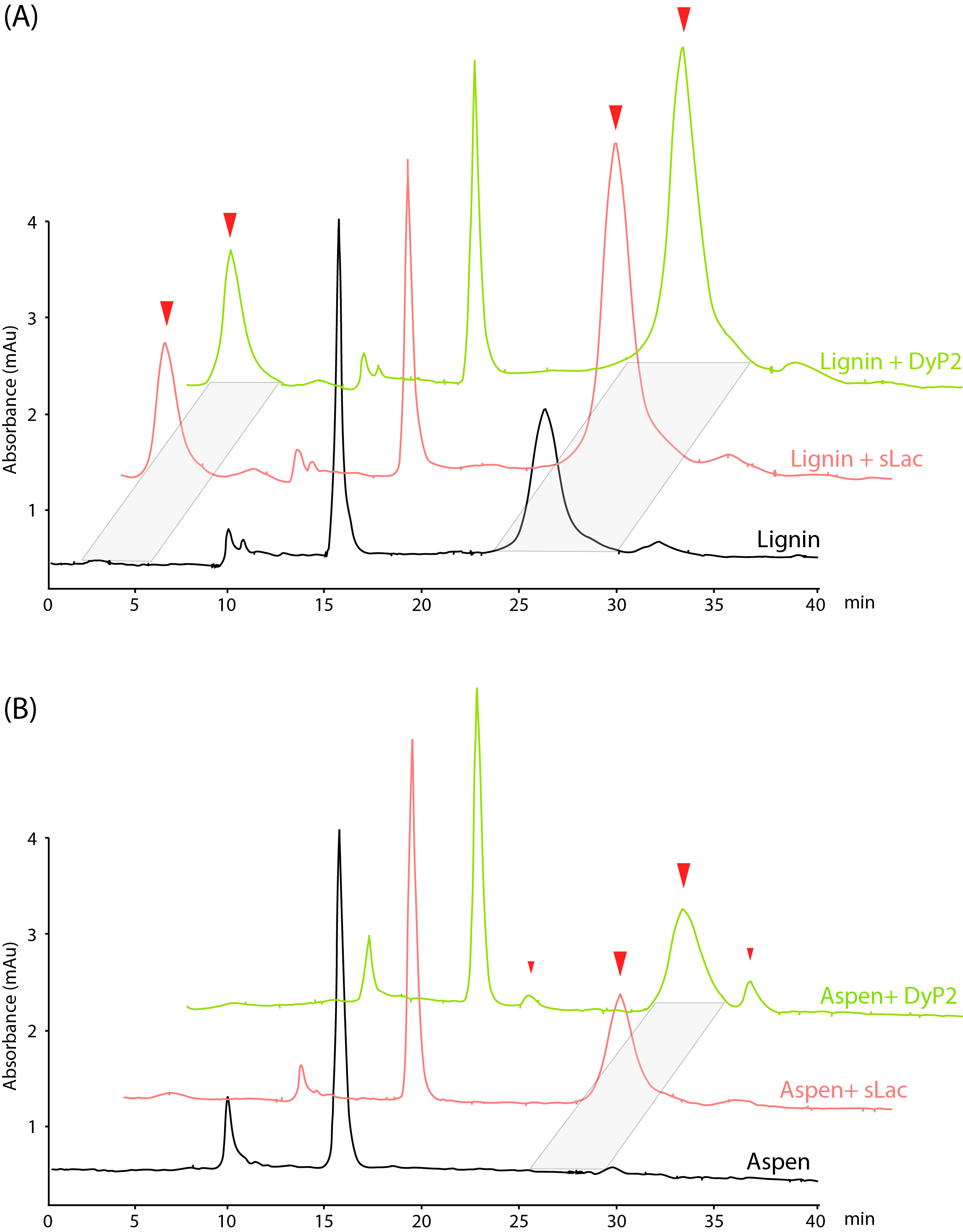


**Figure S4. Soluble products released by sLac and DyP2 from organosolv lignin (A) and aspen wood powder (B)**. The reactions were vacuum-filtered via 1.2 μm, then the filtrates were analyzed by HPLC-UV using an Aminex HPX-87H column and an absorbance detection of 254 nm. Buffer peaks were eluted at 10 min and 15 min. New peaks or peaks with increased signals were labeled by red triangles.

**__**

**Figure S5. Existing amount of H_2_O_2_ in organosolv lignin and aspen wood powder.** Hydrogen peroxide was measured using the Amplex® Red/ horseradish peroxidase (HRP) assay kit for 3 mg of each substrate. Potassium iodide (KI, 20 mM) was also added to decompose existing H_2_O_2_ before measurement of fluorescence.


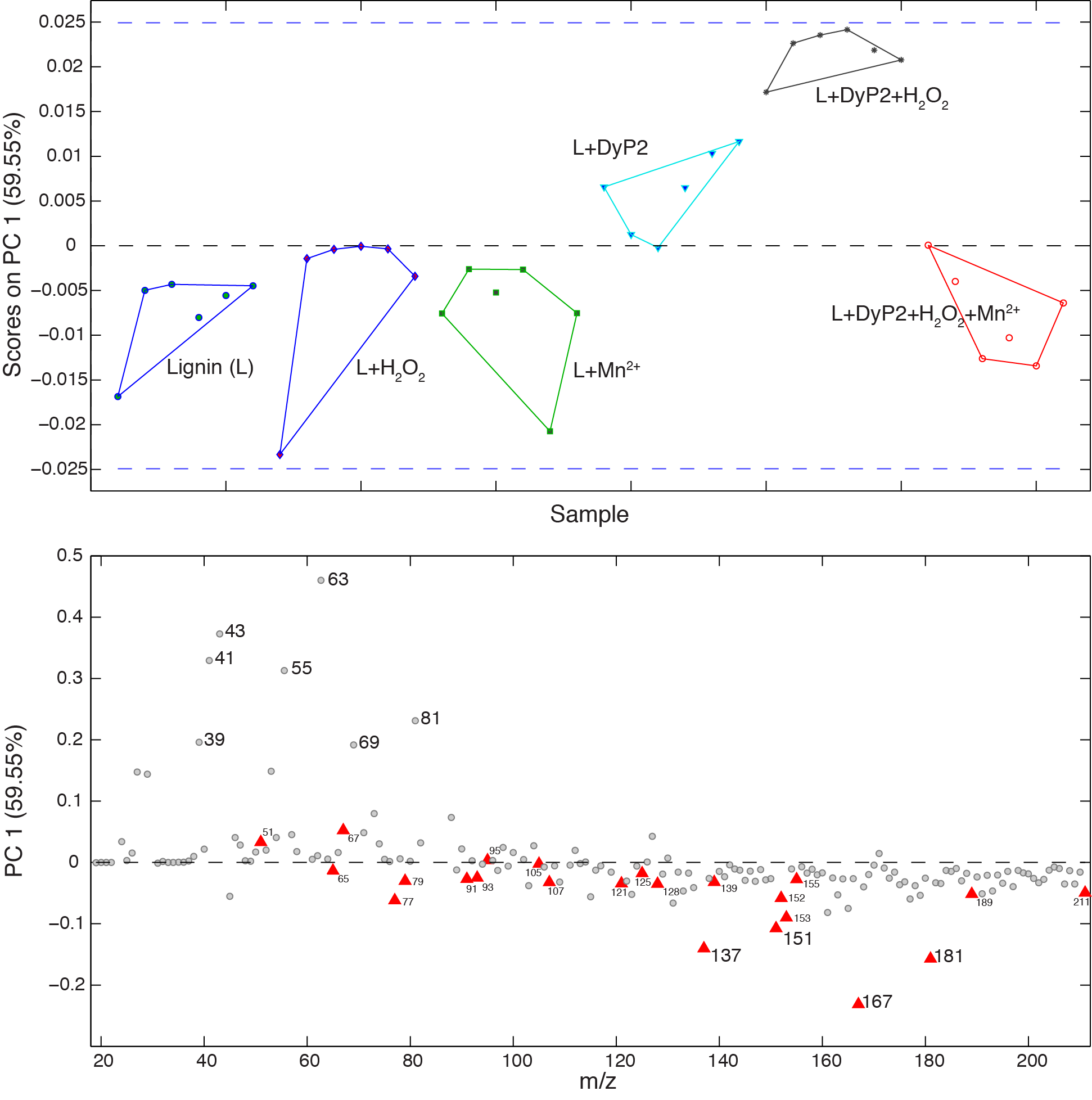


**Figure S6. Addition of Mn^2+^ did not distinguish the sample from the other controls in the experiment of DyP2 on organosolv lignin.** PC1 score (top) and PC1 loading (bottom) of six lignin treatments. Lignin peaks were highlighted as red triangles in the PC1 loading chart. Boundaries of ToF-SIMS acquisition data (n=6) of each treatment were shown by lines.


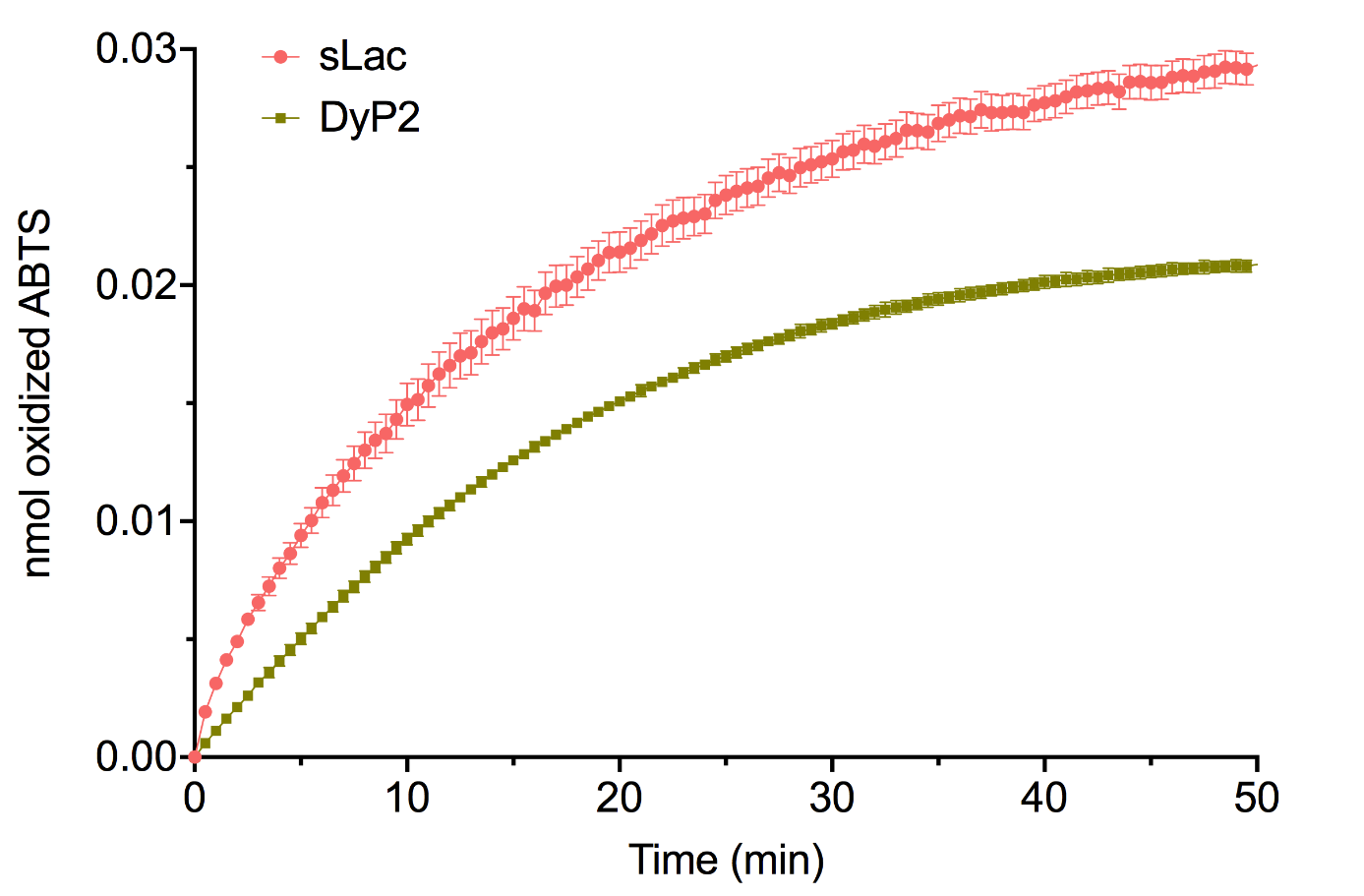


**Figure S7. Specific activity of sLac and DyP2 on ABTS**. Both enzymes (50 μg/mL sLac and 0.4 μg/mL DyP2) were incubated with 0.1 mM ABTS in 50 mM sodium malonate pH 4.5 at 25 °C in the reaction volume of 200 μL. H_2_O_2_ (0.1 mM) was also added in the assay of DyP2. The change in absorbance at 420 nm was measured continuously for 50 min.


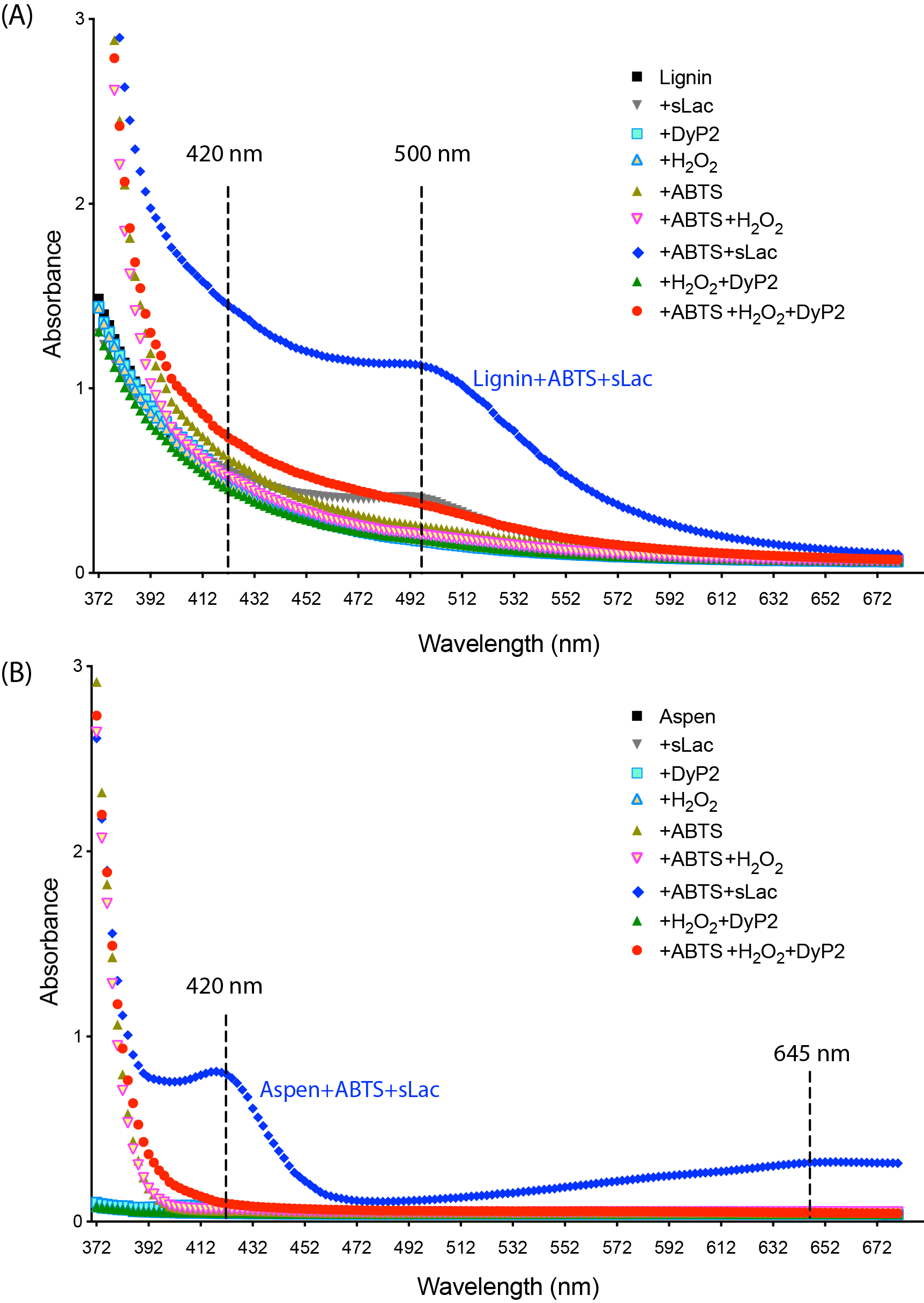


**Figure S8. sLac oxidized ABTS in the presence of organosolv lignin and aspen wood powder**. Absorbance scans of the flow-through after incubation of sLac and DyP2 with organosolv lignin (A) and aspen wood powder (B). Oxidized ABTS products showed absorbance at 420 nm and 645 nm while a new peak was shown at 500 nm.


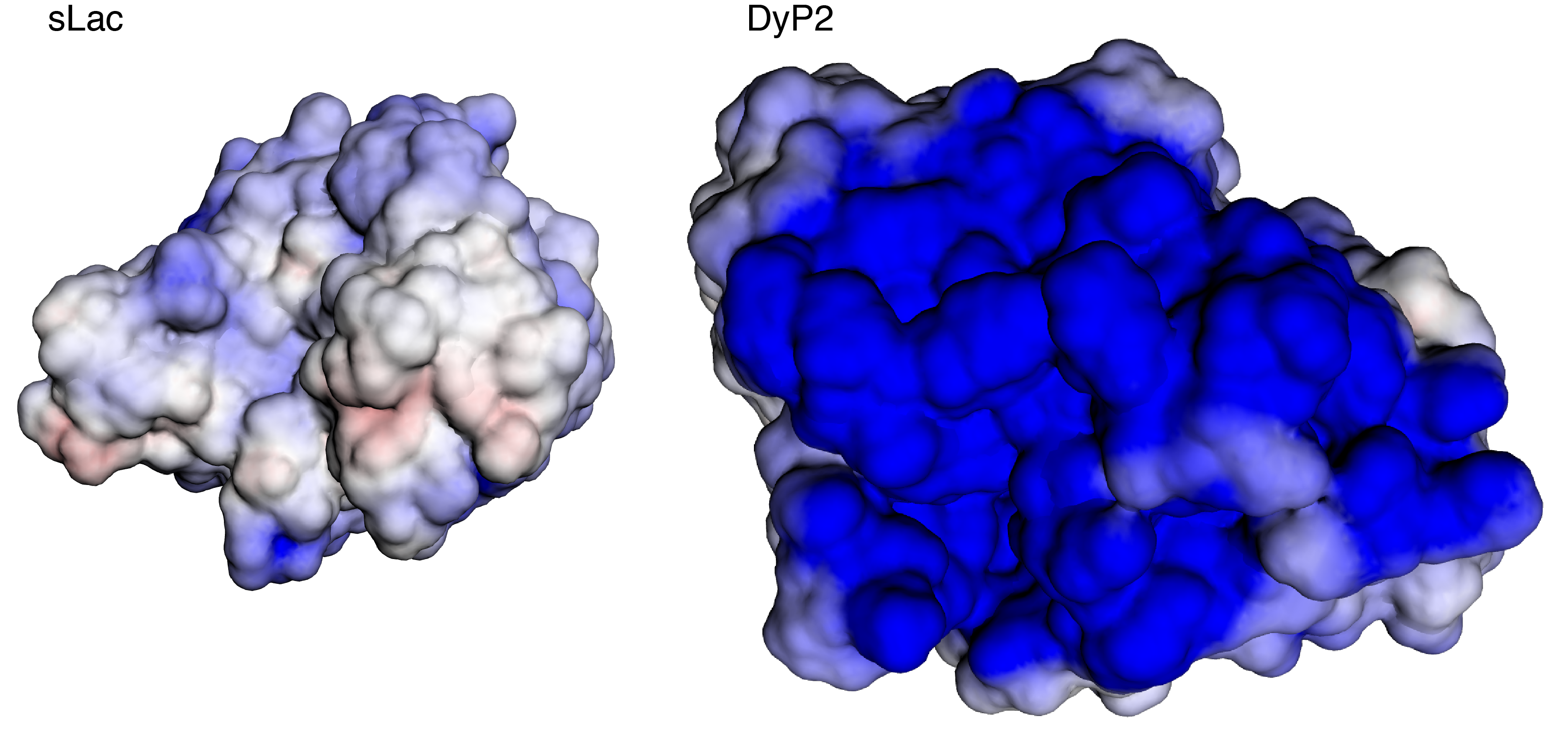


**Figure S9. Electrostatic surface properties of sLac and DyP2**. The x-ray structures of sLac (PDB ID: 3t9w) and DyP2 (PDB ID: 4g2c) were analyzed by the Adaptive Poisson-Boltzmann Solver (https://server.poissonboltzmann.org). Only the monomeric forms of sLac (31 kDa) and DyP2 (50 kDa) were used. The negatively-charged surface was shown in red while the positively-charged surface was in blue.
